# Tracheostomy in the coronavirus disease 2019 patient: evaluating feasibility, challenges and early outcomes of the 14-day guidance

**DOI:** 10.1017/S0022215120001759

**Published:** 2020-08-06

**Authors:** N Glibbery, K Karamali, C Walker, I Fitzgerald O'Connor, B Fish, E Irune

**Affiliations:** Department of Otolaryngology, Head and Neck Surgery, Addenbrooke's Hospital, Cambridge, UK

**Keywords:** Tracheostomy, Coronavirus Disease 2019, SARS-CoV-2, Critical Care, Personal Protective Equipment, Multidisciplinary Team

## Abstract

**Objectives:**

To report feasibility, early outcomes and challenges of implementing a 14-day threshold for undertaking surgical tracheostomy in the critically ill coronavirus disease 2019 patient.

**Methods:**

Twenty-eight coronavirus disease 2019 patients underwent tracheostomy. Demographics, risk factors, ventilatory assistance, organ support and logistics were assessed.

**Results:**

The mean time from intubation to tracheostomy formation was 17.0 days (standard deviation = 4.4, range 8–26 days). Mean time to decannulation was 15.8 days (standard deviation = 9.4) and mean time to intensive care unit stepdown to a ward was 19.2 days (standard deviation = 6.8). The time from intubation to tracheostomy was strongly positively correlated with: duration of mechanical ventilation (r(23) = 0.66; *p* < 0.001), time from intubation to decannulation (r(23) = 0.66; *p* < 0.001) and time from intubation to intensive care unit discharge (r(23) = 0.71; *p* < 0.001).

**Conclusion:**

Performing a tracheostomy in coronavirus disease 2019 positive patients at 8–14 days following intubation is compatible with favourable outcomes. Multidisciplinary team input is crucial to patient selection.

## Introduction

In late December 2019, health authorities in Wuhan City, China, reported the emergence of a novel human coronavirus infection,^1^ which was subsequently named coronavirus disease 2019 (Covid-19) by the World Health Organization (WHO).^2^ The causative pathogen has since been identified as severe acute respiratory syndrome coronavirus-2 (SARS-CoV-2),^1,2^ now at the heart of a major international outbreak.

Coronaviruses are zoonotic pathogens that have been associated with a number of infectious disease outbreaks in humans, including severe acute respiratory syndrome in 2002–2003 and Middle East respiratory syndrome (‘MERS’) in 2012.^3^ Whilst the novel SARS-CoV-2 is a beta coronavirus very similar to the aforementioned severe acute respiratory syndrome,^4–6^ it appears to be associated with higher transmissibility.^3^ On 11th March 2020, WHO officially declared the Covid-19 outbreak a global pandemic, given the rate and extent of its dissemination.^7^

Patients infected with SARS-CoV-2 can be asymptomatic carriers or present with a variety of symptoms. Symptoms range from a mild upper respiratory tract infection to severe viral pneumonia and respiratory failure.^8,9^ Whilst most patients experience mild and self-limiting symptoms, the disease can lead to death in approximately 3–4 per cent of cases.^10^

The outbreak of SARS-CoV-2 has led to a sharp rise in intensive care unit admissions,^11^ with up to 15 per cent of infected individuals requiring critical care because of severe respiratory failure.^12,13^ The surge in intensive care unit admissions and need for prolonged mechanical ventilation has, in turn, led to an increase in the requirement for tracheostomies.^14^

Traditionally, a tracheostomy is performed to aid weaning from ventilatory support, facilitate clearance of secretions, and prevent complications of prolonged intubation such as subglottic stenosis and hospital-acquired pneumonia.^14–16^ However, the role and safety of tracheostomies in patients with Covid-19 has been the source of great debate recently, with very limited experiential information available in the literature. The number of challenges associated with performing tracheostomies in patients with Covid-19 has led to the recent publication of numerous guidelines and recommendations worldwide.^17^

Severe acute respiratory syndrome coronavirus-2 can be transmitted via a number of routes including droplets, fomites and aerosols,^18^ with a high viral load present in sputum and upper airway secretions.^19^ Tracheostomy formation is considered an aerosol-generating procedure, hence posing an infection risk to those healthcare professionals involved intra-operatively.^19,20^ Thus, the highest level of personal protective equipment (PPE) is required when undertaking a tracheostomy, to minimise infection risk. This brings with it the difficulties of resource planning because of the scarcity of said equipment. A further challenge lies in determining patient suitability, likelihood of favourable outcomes and appropriate timing for tracheostomy formation. The Intensive Care National Audit and Research Centre report released on 29th May 2020 indicated that the mortality rate for patients admitted to critical care with Covid-19 requiring advanced respiratory support is 52.4 per cent.^21^

On 19th March 2020, the British Association of Otorhinolaryngology, Head and Neck Surgery (ENT UK) released the first set of recommendations for undertaking tracheostomies during the Covid-19 pandemic.^22^ Subsequently, guideline documents have also been published by the British Laryngological Association and the National Tracheostomy Safety Project.^23,24^ These guidelines aim to provide a decision-making framework for surgeons during unprecedented times, where evidence-based knowledge in this patient group is limited.

This paper aims to discuss our experience in performing surgical tracheostomies during the Covid-19 pandemic at our institution, applying the proposed guidance of the time.^22–24^ We describe the evolution of our clinical practice, which involved the creation of a new tracheostomy multidisciplinary team (MDT), and the early outcomes of Covid-19 infected patients undergoing a surgical tracheostomy.

## Materials and methods

We conducted a prospective institutional review of adult patients admitted to the intensive care unit between 15th March and 20th May 2020 with confirmed Covid-19 and respiratory failure requiring mechanical ventilation. Patients who underwent a surgical or percutaneous tracheostomy for weaning off mechanical ventilation were included in the analysis. The study was conducted at a UK tertiary referral centre (Addenbrooke's Hospital, Cambridge) and was approved by the Trust's clinical audit department prior to data publication.

The electronic medical records of the patients deemed eligible for inclusion were reviewed. The following data were collected: patient demographics, co-morbidities, Acute Physiology and Chronic Health Evaluation II (‘APACHE II’) score, Covid-19 status, respiratory and organ support requirements during intensive care unit admission, tracheostomy procedural details, intra- and post-operative complications, intensive care unit outcomes, and mortality.

Coronavirus disease 2019 status was determined by performing SARS-CoV-2 polymerase chain reaction testing on oropharyngeal or nasopharyngeal swabs, or on sputum or bronchoalveolar lavage samples. Respiratory and organ support requirements on the morning of the tracheostomy procedure (between 6am and 9am) were reviewed.

The parameters assessed included: positive end-expiratory pressure, fraction of inspired oxygen requirements, partial pressure of oxygen/fraction of inspired oxygen ratio, and need for inotropic support or renal replacement therapy. Respiratory support requirements on day 1 and day 7 of mechanical ventilation, as well as on days 5 and 7 post-tracheostomy, are also included in the results. Tracheostomy intra-operative details are described, including level of PPE used and modifications in surgical technique. The infection risk to staff involved in the procedures was also captured.

Our study also explored the early outcomes of intensive care unit patients post-tracheostomy. This included details on the timing of: weaning from intravenous sedation, weaning from mechanical ventilation, successful decannulation, intensive care unit discharge to a general ward, and hospital discharge. Complications, such as a return to the operating theatre, failed decannulation, intensive care unit re-admission and death, were recorded.

Descriptive data on the above parameters are presented, analysed and discussed. Statistical analyses were performed using IBM SPSS® software version 26.

## Results

From 15th March 2020 to 20th May 2020, Addenbrooke's Hospital admitted 97 adult patients with Covid-19 who required critical care support. The majority of these patients required mechanical ventilation for severe respiratory failure.

Twenty-five SARS-CoV-2 positive (Covid-19) patients who underwent a surgical tracheostomy and three patients who underwent a percutaneous tracheostomy, for weaning from mechanical ventilation, were included in the data analysis. Intensive care unit patients without Covid-19 requiring tracheostomy were excluded from our sample population. The commonest co-morbidities reported in the medical notes included hypertension, diabetes mellitus, and respiratory and cardiac disease. A summary of the patient demographics and co-morbidities is included in Table 1.

**Table 1.** Demographics and co-morbidities of patients with Covid-19 undergoing tracheostomy*

Demographics	Values
Age (years)	25–82
– Mean (SD)	60.5 (12.4)
– Median (IQR)	62.5 (18.6)
Sex (*n* (%))	
– Male	20 (71.4)
– Female	8 (28.6)
Ethnicity (*n* (%))	
– White	21 (75.0)
– Black	1 (3.6)
– Not recorded	6 (21.4)
BMI (in kg/m^2^) (*n* (%))	
– Underweight (BMI <18.5)	0 (0.0)
– Normal weight (BMI = 18.5–24.9)	3 (10.7)
– Overweight (BMI = 25–29.9)	9 (32.1)
– Moderately obese (BMI = 30–34.9)	7 (25.0)
– Severely obese (BMI = 35–39.9)	5 (17.9)
– Very severely obese (BMI ≥40)	4 (14.3)
Smoking status (*n* (%))	
– Never smoked	14 (50.0)
– Ex-smoker	10 (35.7)
– Current smoker	1 (3.6)
– Not recorded	3 (10.7)
Co-morbidities (*n* (%))	
– Asthma	5 (17.9)
– COPD	2 (7.1)
– Other respiratory	1 (3.6)
– Hypertension	12 (42.9)
– Other cardiac	4 (14.3)
– Immunosuppression	1 (3.6)
– Renal disease	3 (10.7)
– Diabetes	8 (28.6)
– Other	9 (32.1)
APACHE II score	
– Mean (SD)	13.5 (3.5)
– Median (IQR)	14 (5)

* *n* = 28. Covid-19 = coronavirus disease 2019; SD = standard deviation; IQR = interquartile range; BMI = body mass index; COPD = chronic obstructive pulmonary disease; APACHE II = Acute Physiology and Chronic Health Evaluation II

All referrals for a tracheostomy were discussed at a novel tracheostomy multidisciplinary team meeting.

**Fig. 1.**
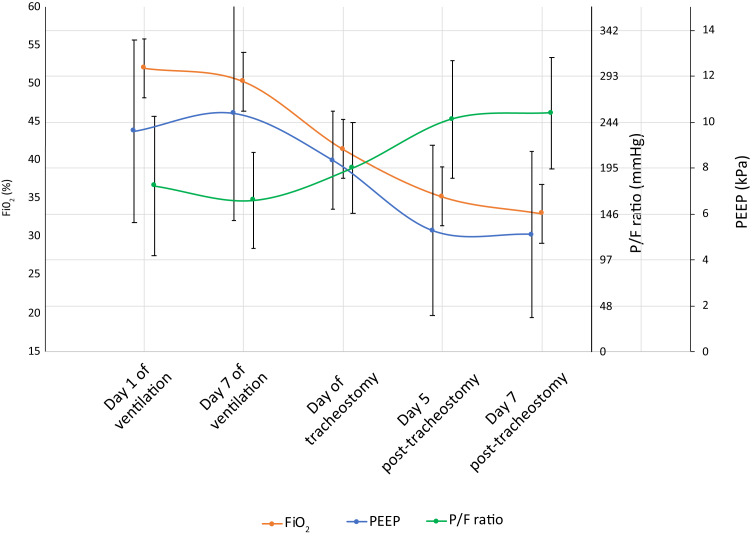
Mean ventilatory requirements (positive end-expiratory pressure, fraction of inspired oxygen, pressure of oxygen/fraction of inspired oxygen ratio) on: days 1 and 7 of mechanical ventilation, on day of tracheostomy, and on days 5 and 7 post-tracheostomy. FiO_2_ = fraction of inspired oxygen; P/F ratio = partial pressure of oxygen/fraction of inspired oxygen; PEEP = positive end-expiratory pressure

The mean time from intubation to tracheostomy formation was at 17.0 days of mechanical ventilation (standard deviation (SD) = 4.4, range of 8–26 days), with 19 tracheostomies (67.9 per cent) performed after day 14. The mean positive end-expiratory pressure at the time of the procedure was 8 mmHg (SD = 2), with fraction of inspired oxygen requirements of 40 per cent (SD = 9). The mean pressure of oxygen/fraction of inspired oxygen ratio was 196 mmHg (SD = 49). Of the 28 patients, 10 (35.7 per cent) were on inotropic support and 12 (42.9 per cent) required renal replacement therapy, with 7 patients (25.0 per cent) having requirements for both. Figure 1 depicts the mean ventilatory requirements, across all patients, on day 1 and day 7 of mechanical ventilation, as well as on days 5 and 7 post-tracheostomy.

Surgical tracheostomies were performed in operating theatres, whereas percutaneous tracheostomies were performed in the intensive care unit. All healthcare workers involved in the procedures wore full PPE, including a filtering facepiece code 3 (FFP3) mask, eye protection, a double-layered fluid-repellent disposable surgical gown and double-layered gloves. Surgeons utilised powered air-purifying respirators.

Adjustments to the surgical tracheostomy technique were made to reduce the aerosolisation of particles and to minimise infection risk. Specifically, the tracheal window was made with the endotracheal tube advanced into the trachea far past the localised site, with the cuff over-inflated. Ventilation was ceased before the endotracheal tube cuff was deflated and the endotracheal tube retracted. The tracheostomy tube was then inserted into the tracheal window and connected to a closed suction system and a viral filter. This high efficiency viral filter was in turn coupled to the ventilator, and only once the circuit was sealed did ventilation re-commence.

The mean procedure time was 97.1 minutes (SD = 22.7), measured from the start of the anaesthetic to the WHO ‘time out’ stage (before skin incision). Cuffed, non-fenestrated tracheostomy tubes were used in all cases, with one patient requiring an adjustable flange. To date, none of the healthcare professionals involved in the tracheostomies intra-operatively have developed clinical symptoms of Covid-19.

No intra-procedural complications were reported. Table 2 summarises tracheostomy-related complications and further intensive care unit related complications in our group of patients, including failed decannulations, unexpected returns to the operating theatre and intensive care unit re-admissions.

**Table 2.** Summary and outcomes of tracheostomy-related post-operative complications, failed decannulations, and ICU re-admissions in Covid-19 patients

Case no.	Tracheostomy	Complication or morbidity
1	Surgical	Inadvertent transection of tracheostomy pilot balloon day 2 post-tracheostomy, requiring re-intubation & revision tracheostomy. Tracheostomy cuff leak day 7 post-op requiring further re-intubation & revision tracheostomy
2	Surgical	Tracheostomy cuff leak post-op requiring re-intubation & revision tracheostomy
3	Surgical	Tracheostomy cuff leak noted intra-op, hence tracheostomy tube replaced. Further cuff leak post-op requiring re-intubation & revision tracheostomy
4	Percutaneous	Bleeding post-tracheostomy, which self-resolved
5	Surgical	Failed decannulation day 3 post-tracheostomy. Subsequent successful decannulation day 6 post-op
6	Percutaneous	Failed decannulation day 34 post-tracheostomy. Subsequent successful decannulation day 44 post-op
7	Surgical	Failed decannulation twice. Required re-intubation because of respiratory failure & repeat tracheostomy. Remains in ICU
8	Surgical	Unplanned re-admission to ICU within 48 h of stepdown because of thick respiratory secretions & intermittent desaturation
9	Surgical	Unplanned re-admission to ICU within 48 h of stepdown because of respiratory deterioration. Condition complicated by development of opsoclonus-myoclonus syndrome
10	Surgical	Unplanned re-admission to ICU within 48 h of stepdown because of respiratory tract infection & desaturation
11	Surgical	Unplanned re-admission to ICU >48 h of stepdown. Development of unexplained stridor & type 2 respiratory failure following blood transfusion, requiring re-intubation. Unclear cause of deterioration. Subsequent successful discharge to general ward
12	Surgical	Unplanned re-admission to ICU >48 h of stepdown because of pericardial effusion development in the context of polytrauma (polytrauma patient with Covid-19)

ICU = intensive care unit; Covid-19 = coronavirus disease 2019; case no. = case number; post-op = post-operatively; intra-op = intra-operatively; h = hours

**Fig. 2.**
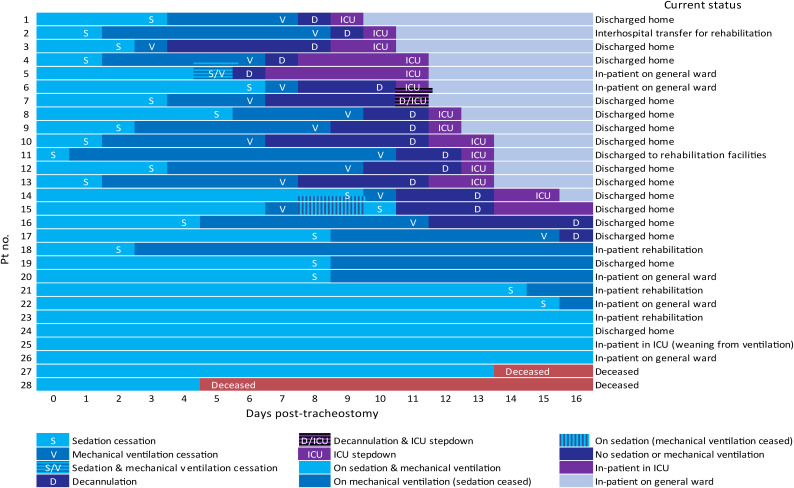
Outcomes of patients during the first 16 days post-tracheostomy and details of their current state. Pt no. = patient number; ICU = intensive care unit

Analysis of our available data showed that the mean time from tracheostomy to weaning from intravenous sedation was 8.2 days (SD = 9.7). The mean time to weaning from mechanical ventilation was 13.4 days (SD = 9.7). The mean time to decannulation was 15.8 days (SD = 9.4). The mean time to intensive care unit stepdown to a general ward was 19.2 days (SD = 10.5). Figure 2 summarises the outcomes in this cohort of patients during their first 16 days post-tracheostomy.

We compared outcomes in the patients who underwent a tracheostomy following 14 days or fewer of mechanical ventilation versus those whose tracheostomy was performed after 14 days of mechanical ventilation. Interestingly, weaning from mechanical ventilation, decannulation and intensive care unit stepdown appeared to be taking place sooner in the subgroup undergoing early tracheostomy formation, following 14 days or fewer of ventilation. In this group, mean time from intubation to weaning from mechanical ventilation, decannulation and intensive care unit discharge was 22.2 days (SD = 5.4), 24.9 days (SD = 4.6) and 27.7 days (SD = 7.2), respectively. In comparison, in those undergoing tracheostomy after 14 days of ventilation, mean time from intubation to weaning from mechanical ventilation, decannulation and intensive care unit discharge was 33.6 days (SD = 12.5), 36.0 days (SD = 12.4) and 39.7 days (SD = 13.4), respectively.

**Fig. 3.**
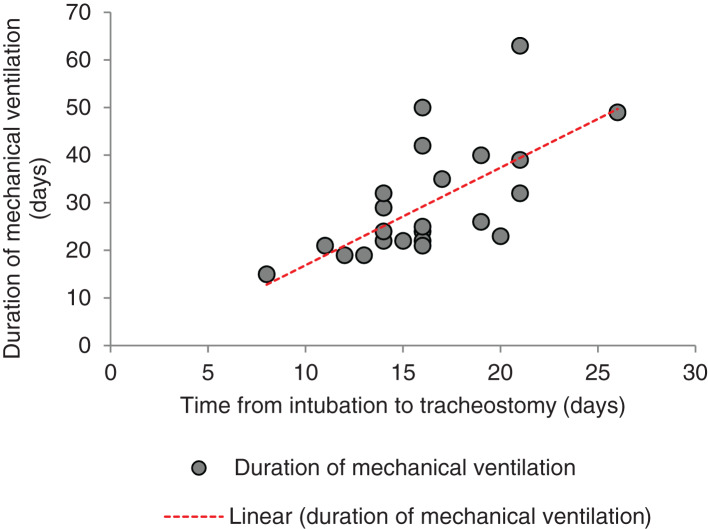
Inter-relationship between duration of mechanical ventilation and time from intubation to tracheostomy (y = 2.0492x – 3.5956; R^2^ = 0.4403).

**Fig. 4.**
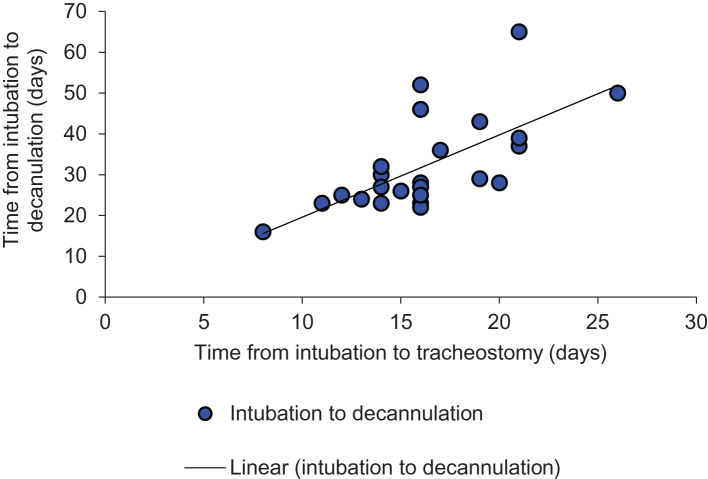
Inter-relationship between time from intubation to decannulation and time from intubation to tracheostomy (y = 2.0151x - 0.5642; R^2^ = 0.4467).

**Fig. 5.**
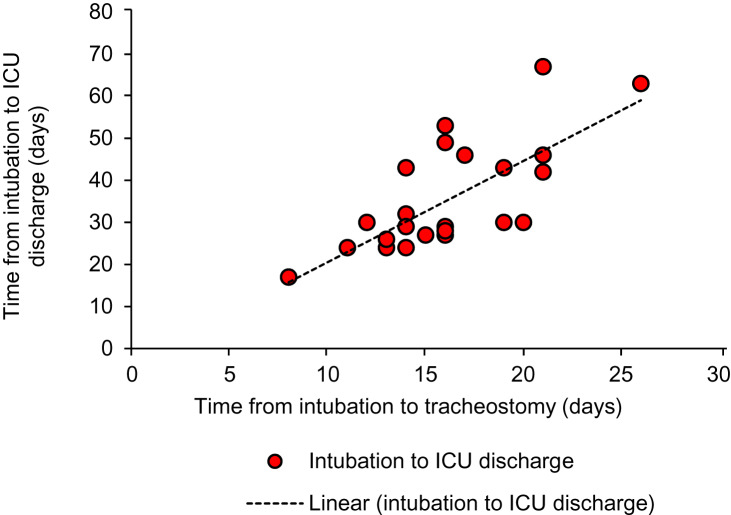
Inter-relationship between time from intubation to intensive care unit (ICU) discharge and time from intubation to tracheostomy (y = 2.4031x - 3.4747; R^2^ = 0.5098).

Statistical analysis was performed using a Pearson correlation (excluding 3 out of the 28 subjects for reasons explained in Figure 2); the results of the analysis support the findings above. The analysis showed strong positive correlations between the time period from intubation to tracheostomy and: the duration of mechanical ventilation (r(23) = 0.66; *p* < 0.001, two-tailed), the time from intubation to decannulation (r(23) = 0.66; *p* < 0.001, two-tailed), and the time from intubation to intensive care unit discharge (r(23) = 0.71; *p* < 0.001, two-tailed) (Figures 3–5).

The above results were further confirmed by a Spearman rank-order correlation. This demonstrated a strong positive correlation between the time from intubation to tracheostomy and all three dependent variables, namely: duration of mechanical ventilation (r = 0.7; *p* < 0.001, two-tailed), time from intubation to decannulation (r = 0.7; *p* < 0.001, two-tailed), and time from intubation to intensive care unit discharge (r = 0.7; *p* < 0.001, two-tailed).

Furthermore, the distributions of age, gender, body mass index (BMI), and Acute Physiology and Chronic Health Evaluation II score, and the pressure of oxygen/fraction of inspired oxygen ratio, in either the ‘early’ tracheostomy group (14 days or fewer of mechanical ventilation) or ‘late’ tracheostomy group (after 14 days of mechanical ventilation), had no impact on the positive correlations demonstrated above. This latter finding is likely influenced by the small sample size and must be interpreted with caution.

In an effort to adjust for potential confounding variables between both groups, we compared demographic information and indicators of acute severity mentioned above. The following variables were included in the analysis (performed using Mann–Whitney U tests, Fisher's exact tests and independent *t*-tests): age (*p* = 0.17), sex (*p* = 1.00), BMI (*p* = 0.31), Acute Physiology and Chronic Health Evaluation II score (*p* = 0.93), pressure of oxygen/fraction of inspired oxygen ratio on admission (*p* = 0.64), and presence of severe co-morbidities (*p* = 0.52) as defined in the Intensive Care National Audit and Research Centre report.^21^ The statistical analysis demonstrated no significant differences between the two groups.

At the time of writing, the mean follow-up time for all the patients after tracheostomy formation was 57.7 days (SD = 11.7), whereas the total follow-up time following intubation was 74.7 days (SD = 11.6). Of the 28 patients who underwent tracheostomy, 1 patient (3.6 per cent) is undergoing weaning from ventilation, 5 (17.9 per cent) are in-patients on general wards, 15 (53.6 per cent) have been discharged home, 4 (14.2 per cent) have been discharged to in-patient rehabilitation facilities and 1 (3.6 per cent) has been repatriated to a local hospital for further rehabilitation. Unfortunately, two patients (7.1 per cent) clinically deteriorated in the intensive care unit and are deceased. These deaths occurred on days 5 and 14 post-tracheostomy.

## Discussion

The ENT UK, British Laryngological Association and National Tracheostomy Safety Project released national recommendations to aid difficult decision-making during these unprecedented, challenging times.^22–24^ The salient points of their recommendations include the following: (1) the appropriateness of tracheostomy in Covid-19 positive patients should be discussed between senior ENT and intensive care unit clinicians; (2) the delaying of tracheostomy until active disease has passed should be considered; (3) tracheostomy is unlikely to be indicated at fewer than 14 days of ventilation; (4) the patient should ideally be apyrexial, with falling inflammatory markers and minimal pressor support; (5) the patient should be requiring a positive end-expiratory pressure of 10 cmH_2_O or less and fraction of inspired oxygen of 0.4 or less; (6) the procedure should be performed by the most skilled anaesthetist and ENT surgeon, reducing any unnecessary team members; (7) full PPE is to be used, including an FFP3 mask, eye and face protection, and a fluid-resistant disposable gown, and ‘double-gloving’ should be considered; (8) a cuffed, non-fenestrated tracheostomy tube should be used; (9) initial advancement of the endotracheal tube should be performed prior to making the tracheostomy window; and (10) a ‘Covid airway team’ should be created and identified within ENT departments.

Our cohort of 28 patients appears to be representative of the intensive care unit patients across the UK, with regard to age, sex, BMI and ethnicity.^21^ Variations are accounted for by our local population characteristics. The commonest co-morbidities in our patient cohort included hypertension, diabetes mellitus, respiratory and cardiac disease. Recent studies have shown similar results. The reported characteristics associated with severe disease and the need for critical care include: male sex, increasing age, high BMI, and the presence of co-morbidities such as hypertension, cardiovascular disease, hypercholesterolaemia and diabetes. A high Acute Physiology and Chronic Health Evaluation II score is known to be associated with adverse outcomes and death.^13,21,25,26^

In our institution, we introduced a tracheostomy MDT meeting, attended by senior intensive care unit physicians, ENT consultants and tracheostomy clinical nurse specialists. This MDT facilitated decision-making regarding tracheostomy in this complex group of patients. It enhanced communication between clinical teams, ensuring the suitability of patients (in terms of co-morbidity and predicted survival), optimal procedural timing (in reference to the severity of acute respiratory distress syndrome and organ support), adequate preparation for the surgical team, and appropriate resource utilisation such as operating theatre time and PPE. Perhaps most importantly, the MDT enabled a shared responsibility for these patients, thus mitigating the moral dilemmas and psychological toll that the clinical management of critically ill Covid-19 patients could pose.

The tracheostomy MDT meeting evolved to become the launch pad for facilitating discussions on the post-intensive care unit journey, informing the deployment of skill and rehabilitation services; for example, supporting challenging decannulations with specialist input, and early referral to speech and language therapists, which led to the recognition and characterisation of post-intubation dysphagia and dysphonia. Another example is the implementation of management strategies, which led to the adoption of technology in ongoing patient care, the development of post-discharge care pathways and opportunities for clinical research. With clinicians and allied healthcare professionals working closely together in the vanguard of clinical care, we gained valuable insight on what was to come. This informed the need to rethink service delivery within our otolaryngology department and secure appropriate funding to support novel clinical amenities.

The decision to undertake percutaneous versus surgical tracheostomy in our cohort of patients was based on two factors: (1) the availability of expertise (as percutaneous tracheostomies are only carried out by intensivists – an already heavily pressurised workforce during the pandemic); and (2) ease of percutaneous accessibility to the trachea. The surgical tracheostomies in our institution were performed only by senior ENT surgeons (working in pairs); this protected other departmental members from unnecessary exposure and facilitated efficient operating. The high standards of PPE made available were predominantly a result of a proactive ENT surgical team and a responsive senior leadership team that took concerns seriously. Team-working with our anaesthetic colleagues, the scrub team, early adoption of a strict protocol in terms of patient scheduling, intra-operative equipment and technique were crucial to delivering a robust service. Processes that safeguard the appropriate donning and doffing of PPE (with a spotting colleague) were adhered to without compromise. Our operating theatre staff were instrumental in implementing appropriate patient flow and to the overall support of the service.

The single undefeated conundrum is that of communication whilst dressed in full PPE. This is further compounded by the powered air-purifying respirator worn by the operating surgeons. This meant surgeons often had to raise their voices and repeat instructions. Most of the fatigue from operating arose from this difficulty in interacting with colleagues, who were unable to read facial expressions, as well as the perpetual delays in transporting patients and working within a surgical team that had more than doubled in size in order to run a complex set up. There is no doubt that the time taken to don and doff PPE significantly extended peri-operative time. There were also delays arising from waiting 20 minutes to allow adequate air exchange following an aerosol-generating procedure before any member of staff could depart the operating theatre suite. These logistical issues are reflected in our longer operating times, averaging at 97.9 minutes and ranging from 70 to 147 minutes, compared to previously documented standard procedure lengths.^27^

Ventilatory and organ support requirements in our patient cohort may be considered higher than the ideal requirements described in the recent national recommendations; however, we posit that the complexity of these cases warranted a case-by-case assessment.

Our results demonstrated no intra-operative complications and only minimal tracheostomy-related post-operative complications. Of the 28 patients who underwent a tracheostomy, 25 (89.3 per cent) have been successfully discharged from the intensive care unit. Five of these patients (17.9 per cent) remain on medical wards, whereas 20 (71.4 per cent) have been discharged home or to in-patient rehabilitation. Comparatively, the mortality rate of Covid-19 patients who underwent tracheostomy was 7.1 per cent. In contrast, within the whole cohort of patients in our intensive care unit, the mortality rate of patients with Covid-19 was 29.0 per cent.

These data suggest that the tracheostomies undertaken were appropriate and were not associated with increased morbidity in this patient cohort. However, such findings are dependent on the careful selection of patients, to ensure optimal outcomes. An outlier in our cohort is a young male patient who was originally admitted to the intensive care unit as a polytrauma case. He was found to be SARS-CoV-2 positive on admission. This, in combination with his multiple injuries, led to a prolonged and complicated recovery course (he remains an in-patient on day 70 at the time of writing). Nonetheless, we consider our patient group to be representative of the complex, severely ill patients with Covid-19 being admitted to intensive care units across the UK. This highlights the importance of individualised assessment and multidisciplinary decision-making in the management of these patients.

Despite controlling for confounding factors, we observed that the duration from intubation to ceasing mechanical ventilation, the time to decannulation and the length of stay in the intensive care unit were reduced in the subgroup undergoing ‘early’ tracheostomy (following 14 days or fewer of mechanical ventilation) compared with the patients who underwent a ‘late’ tracheostomy (after 14 days of ventilation). This suggests that early tracheostomy might be associated with improved outcomes in patients admitted to the intensive care unit with Covid-19. Though we present these findings with statistical significance, it is important to note that our cohort is of a small sample size. Moreover, the timing and decision to proceed with a tracheostomy is determined by additional variables, including: the patients’ clinical condition, the extent of organ support required, the expected recovery, and logistical issues such as operating theatre and staff availability.

- This paper addresses the feasibility of implementing the 14-day tracheostomy threshold in a regional centre
- It discusses the importance of a multidisciplinary team in patient selection and the journey following intensive care
- The paper echoes the potential for benefits with regard to early tracheostomy
- It discusses the logistics of undertaking tracheostomy, including communication difficulties, personal protective equipment, prolonged procedure timings and scheduling
- It raises the mental health effect and psychological toll of managing coronavirus disease 2019 patients, and the benefits of shared decision-making

Nonetheless, Arabi *et al*. demonstrated that time to tracheostomy was an independent predictor of mechanical ventilation duration, and intensive care unit and hospital admission durations.^28^ Recent systematic reviews have also suggested that early tracheostomy might be associated with reduced intensive care unit length of stay, although its effect on overall intensive care unit mortality is debated.^29,30^ The more recent findings of Angel *et al*.,^31^ based on 98 patients in New York who underwent percutaneous tracheostomy, further support early tracheostomy in the critically ill Covid-19 patient.

## Conclusion

Our preliminary outcomes provide a useful indication of the utility and safety of tracheostomies in this patient cohort. Our data, so far, suggest that utilising the current guidelines as a ‘framework’ is compatible with favourable outcomes, and is associated with low patient morbidity and mortality. There is evidence to suggest that performing an early tracheostomy, following 14 days or fewer of ventilation, might be associated with improved outcomes. However, further work is required to determine whether overall survival can be influenced by this practice. Most importantly, due consideration must be given to the virulence of the SARS-CoV-2 pathogen in the early days of severe respiratory illness, and the potential for transmission to medical personnel.

Surgical tracheostomy in the Covid-19 patient will benefit from multidisciplinary input. Decisions should be made on an individualised basis, to optimise outcomes. A tracheostomy MDT also provides an invaluable platform upon which to build the post-intensive care unit patient treatment and rehabilitation plan. Furthermore, it is likely that the mental health effects of this pandemic on healthcare workers will begin to manifest in the coming months. We postulate that complex MDTs such as this assuage this effect by affording clinicians the opportunity to share the burden of life and death decisions.

Clinical and scientific evidence relating to surgical tracheostomy in the Covid-19 patients is rapidly evolving. By reporting our experience, we endeavour to enable cogent clinical reasoning amongst colleagues facing the same multifarious dilemma: when, where and how, and is it worth the risks?
